# Severe hepatotoxicity due to osimertinib after nivolumab therapy in patients with non‐small cell lung cancer harboring *EGFR* mutation

**DOI:** 10.1111/1759-7714.13363

**Published:** 2020-02-18

**Authors:** Ou Yamaguchi, Kyoichi Kaira, Tomonori Kawasaki, Atsuto Mouri, Kosuke Hashimoto, Ayako Shiono, Shun Shinomiya, Yu Miura, Fuyumi Nishihara, Yoshitake Murayama, Kunihiko Kobayashi, Satoshi Mochida, Hiroshi Kagamu

**Affiliations:** ^1^ Department of Respiratory Medicine, Comprehensive Cancer Center Saitama Medical University International Medical Center Saitama Japan; ^2^ Department of Pathology Saitama Medical University International Medical Center Saitama Japan; ^3^ Department of Gastroenterology and Hepatology, Faculty of Medicine Saitama Medical University Saitama Japan

**Keywords:** Hepatotoxicity, immune checkpoint inhibitor, nivolumab, non‐small‐cell lung cancer, osimertinib

## Abstract

**Background:**

Osimertinib is the most promising treatment option for patients with epidermal growth factor receptor (*EGFR*) mutation‐positive non‐small cell lung cancer (NSCLC) with acquired T790M resistance. However, recent studies have suggested that osimertinib could increase the frequency of serious adverse events (AEs) if administered immediately after immune checkpoint inhibitor (ICI) treatment.

**Methods:**

In this single‐institution retrospective study conducted from May 2016 to January 2019, osimertinib was administered to 47 patients with pretreated advanced NSCLC harboring the *EGFR* mutation.

**Results:**

Of the 47 patients, 20 (42.6%) were men and 27 (57.4%) were women. The median age was 71 years (range 37–83 years). A total of 19 patients (40.4%) had a smoking history. Furthermore, seven patients (14.9%) received osimertinib immediately after nivolumab therapy, while 40 patients (85.1%) were treated with osimertinib after treatment with drugs other than nivolumab. The frequency of grade 3 or 4 hepatotoxicity was significantly higher in patients with nivolumab prior to osimertinib (4/7; 57.1%) than in those treated with drugs other than nivolumab prior to osimertinib (2/40; 5.0%) (*P* = 0.0026). Liver biopsies were performed in two patients who received osimertinib immediately after nivolumab. In both patients, CD‐8‐positive T cell infiltration was predominantly observed in the liver tissues.

**Conclusions:**

The use of osimertinib immediately after nivolumab significantly increased the frequency of grade 3 or higher hepatotoxicity in patients with advanced NSCLC harboring *EGFR* mutation acquired T790M resistance.

## Introduction

Osimertinib is the most promising tyrosine‐kinase inhibitor (TKI) to treat T790M‐acquired resistance in patients with non‐small cell lung cancer (NSCLC) harboring the epidermal growth factor receptor (*EGFR)* mutation.^1^ Immune checkpoint inhibitors (ICIs), such as anti‐programmed death‐1 (PD‐1)/anti‐programmed death ligand‐1 (PD‐L1) antibodies, have been identified as therapeutic agents that may influence long‐term prognosis of patients with NSCLC.^2^ However, combined or sequential use of ICI and EGFR‐TKI is known to potentially increase the risk of known adverse events. Recently, Ahn *et al*. reported that the combination of osimertinib and durvalumab was not appropriate for patients with advanced *EGFR* mutation‐positive NSCLC due to the increased incidence of interstitial lung disease (ILD).^3^ Although only 34 patients were treated with this combination therapy in their study, ILD was observed in 38% of all patients and 60% of Japanese patients.^3^ Moreover, a recent study described an increased incidence of ILD in patients who received osimertinib immediately after nivolumab, an anti‐PD‐1 antibody.^4^, ^5^

Generally, hepatotoxicity is a major adverse event (AE) of anticancer drugs. However, little is known about the incidence of hepatotoxicity accompanying osimertinib administration immediately after ICI treatment. Hepatotoxicity is a common adverse event after the administration of an EGFR‐TKI. The severity of adverse event seems to be stronger with gefitinib than with afatinib or osimertinib. However, drug‐induced hepatotoxicity improves immediately after the cessation of the related agent, although a severe adverse event may occur. On the contrary, drug‐induced hepatotoxicity occurs due to nivolumab, but it is identified as a low incidence. Ahn *et al*. reported the high incidence of drug‐induced lung toxicity associated with the combination of osimertinib and durvalumab; however, there is no report about the occurrence of severe hepatotoxicity due to its combination.^3^ Little is known whether osimertinib after ICI treatment could cause serious hepatotoxicity or increase the incidence of hepatotoxicity. Therefore, additional studies to determine whether sequential use of osimertinib after ICI increases hepatotoxicity risk and ILD are needed. It is difficult to assess the tolerability of osimertinib after ICI treatment regarding hepatotoxicity using a prospective study. Therefore, in this retrospective study, we aimed to characterize serious hepatotoxicity due to osimertinib after nivolumab treatment in patients with previously treated NSCLC harboring the *EGFR* mutation.

## Methods

### Patient and treatment information

We retrospectively examined patients who were histologically or cytologically proven to have NSCLC with *EGFR* T790M‐acquired resistance and received osimertinib after disease progression with first‐ or second‐generation EGFR‐TKI treatment. Patients received 80 mg osimertinib orally once daily. Treatment continued until the progression of the disease, the development of unacceptable AEs, or requested by either the patient or physician to discontinue treatment. Acute toxicity was graded according to the Common Terminology Criteria for Adverse Events version 4.0. Tumor response was evaluated according to response evaluation criteria in solid tumors version 1.1.^6^ We defined our “immediate administration” as “osimertinib administration within 180 days after the last dose of nivolumab”. This study was a single‐institution retrospective study approved by the Institutional Review Board (approval number 19‐062) of Saitama Medical University International Medical Center (SMUIMC).

### Immunohistochemical staining

Immunohistochemical staining was performed to detect CD4‐ (1:200 dilution; Dako, Tokyo, Japan), CD8‐ (1:1000 dilution; Abcam, Tokyo, Japan), CD3‐ (1:200 dilution; Abcam), and CD20‐positive (1:200 dilution; Abcam) cells in the liver specimens. After specimen evaluation, CD4(+), CD8(+), CD3(+), and CD20(+) cells were counted in a selected area under 400 × magnification (0.26 mm^2^ of field area). The tissue sections were examined in a blinded fashion by at least two investigators using a light microscope.

### Statistical analysis

Statistical significance was indicated by *P* < 0.05. Fisher's exact test or chi‐square test was used to examine the association between two categorical variables. Statistical analyses were performed using JMP 8.0 (SAS Institute Inc., Cary, NC, USA).

## Results

### Patient demographics

From May 2016 to January 2019, 51 consecutive patients with *EGFR* T790M‐acquired resistance were treated with osimertinib. Of the 51 patients, four were excluded from this study due to their participation in a clinical trial. Therefore, 47 patients were finally eligible, and patient characteristics are listed in Table 1. A total of 20 patients (42.6%) were men and 27 (57.4%) were women. The median age was 71 years (range 37–83 years). A total of 19 patients (40.4%) had a smoking history. Of the 47 patients, seven patients (14.9%) received osimertinib immediately after the cessation of nivolumab and 40 patients (85.1%) were treated with other agents between osimertinib and nivolumab administration. Therefore, we divided the patients into two groups: the direct sequence group (DSG) which included patients who received osimertinib immediately after cessation of nivolumab, and the nondirect sequence group (non‐DSG) which included patients who were treated with other agents between osimertinib and nivolumab. Further analysis was performed according to the category of DSG and non‐DSG. The percentage of males in the DSG and non‐DSG groups was 85.7% (6/7) and 35.0% (14/40), respectively, with statistical significance (*P* = 0.032). The percentage of patients with the Eastern Cooperative Oncology Group performance score (PS) of 0‐1 in the DSG and non‐DSG groups was 85.7% (6/7) and 80.0% (32/40), respectively, without statistical significance. The percentage of early treatment line (second‐ or third‐line) in the DSG and non‐DSG groups was 0% (0/7) and 52.5% (21/40), respectively, with statistical significance (*P* = 0.012).

**Table 1:** Demographics of osimertinib‐treated patients

Variable (*n* = 47)	All patients treated with osimertinib			
	(*n* = 47)	Prior treatment		*P*‐value
		DSG (*n* = 7)	non‐DSG (*n* = 40)	
Age				
<75/≥ 75 years	33/14	5/2	28/12	1.000
Gender				
Men/Women	20/27	6/1	14/26	0.032
Smoking				
Yes/No	19/28	4/3	15/25	0.417
ECOG PS				
0–1/2–4	38/9	6/1	32/8	1.000
Type of *EGFR* mutation				
Exon19del/L858R/others	30/16/1	3/4/0	28/11/1	0.289
Means of T790M detection				
Liquid/other method	8/39	2/5	6/34	0.585
Treatment line of osimertinib				
Second or third/over fourth	21/26	0/7	21/19	0.012

DSG, direct sequence group; ECOG, Eastern Cooperative Oncology Group; EGFR, epidermal growth factor receptor; non‐DSG, nondirect sequence group; PS, performance status.

### Hepatotoxicity after administration of nivolumab

Grade 3 or 4 hepatotoxicity was significantly observed in four of the seven patients (57.1%) with DSG and two of the 40 patients (5.0%) with non‐DSG, respectively (*P* = 0.0026).

Table 2 shows a list of patients who presented grade 3 or 4 transaminase level elevation. Furthermore, a liver biopsy was performed in two of the four patients with DSG (Cases 2 and 4). The time until onset of grade 3 transaminase level elevation tended to be longer in patients with DSG than that in patients with non‐DSG. Liver dysfunction was persistent in patients with DSG compared with that in patients with non‐DSG; thus, immunosuppressive therapy such as steroids was required.

**Table 2:** Characteristics of patients with hepatotoxicity due to osimertinib

Variable	Case 1	Case 2	Case 3	Case 4	Case 5	Case 6
Age (years)	75	77	59	63	71	74
Gender	Men	Men	Men	Men	Men	Female
Treatment line	7th	7th	7th	6th	2nd	5th
Pretreatment	Nivolumab	Nivolumab	Nivolumab	Nivolumab	Erlotinib	Docetaxel
Best response of nivolumab	PD	SD	PR	PD	‐	‐
Time from last nivolumab administration to start of osimertinib, days	36	180	24	32	‐	‐
Time from start of osimertinib to CTCAE grade three transaminase elevation, days	46	526	35	29	9	14
Liver metastasis at start of osimertinib	None	None	None	Present	None	None
AST, max, U/dL	214	150	905	370	244	78
ALT, max, U/dL	90	236	688	453	501	222
Liver biopsy	None	Done	None	Done	None	None
Steroid treatment for hepatotoxicity	None	Done, prednisolone 40 mg/day	None	Done, prednisolone 120 mg/day	None	None
Outcome of hepatotoxicity	Improved	Improved	Improved	Improved	Improved	Improved
Best response of osimertinib	PR	PR	PR	PR	NE	PR
Other AEs with osimertinib		Pneumonitis	Pneumonitis			

ALT, alanine aminotransferase; AST, aspartate aminotransferase; CTCAE, Common Terminology Criteria for Adverse Events; NE, not evaluable; PD, progressive disease; PR, partial response; SD, stable disease.

### Improvements of liver dysfunction in patients after osimertinib treatment

**Figure 1:**
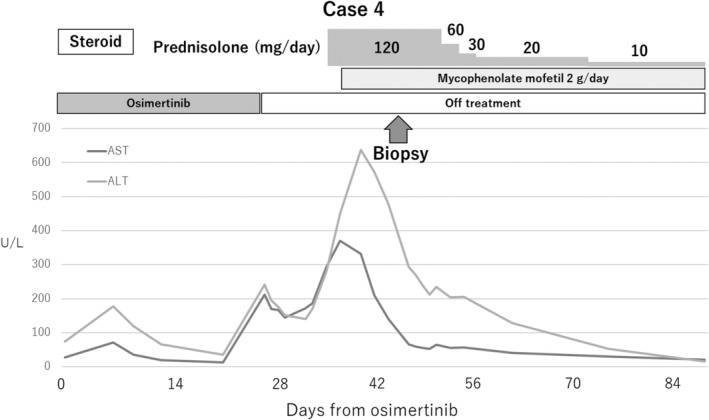
Clinical course of Case 4. Grade 3 transaminase elevation was observed at 24 days after the start of osimertinib treatment. A liver biopsy was performed following steroid therapy to investigate the cause of liver dysfunction. Mycophenolate mofetil was additionally administered due to transient transaminase elevation after prednisolone initiation. Transaminase levels slowly decreased, and then normalized.

**Figure 2:**
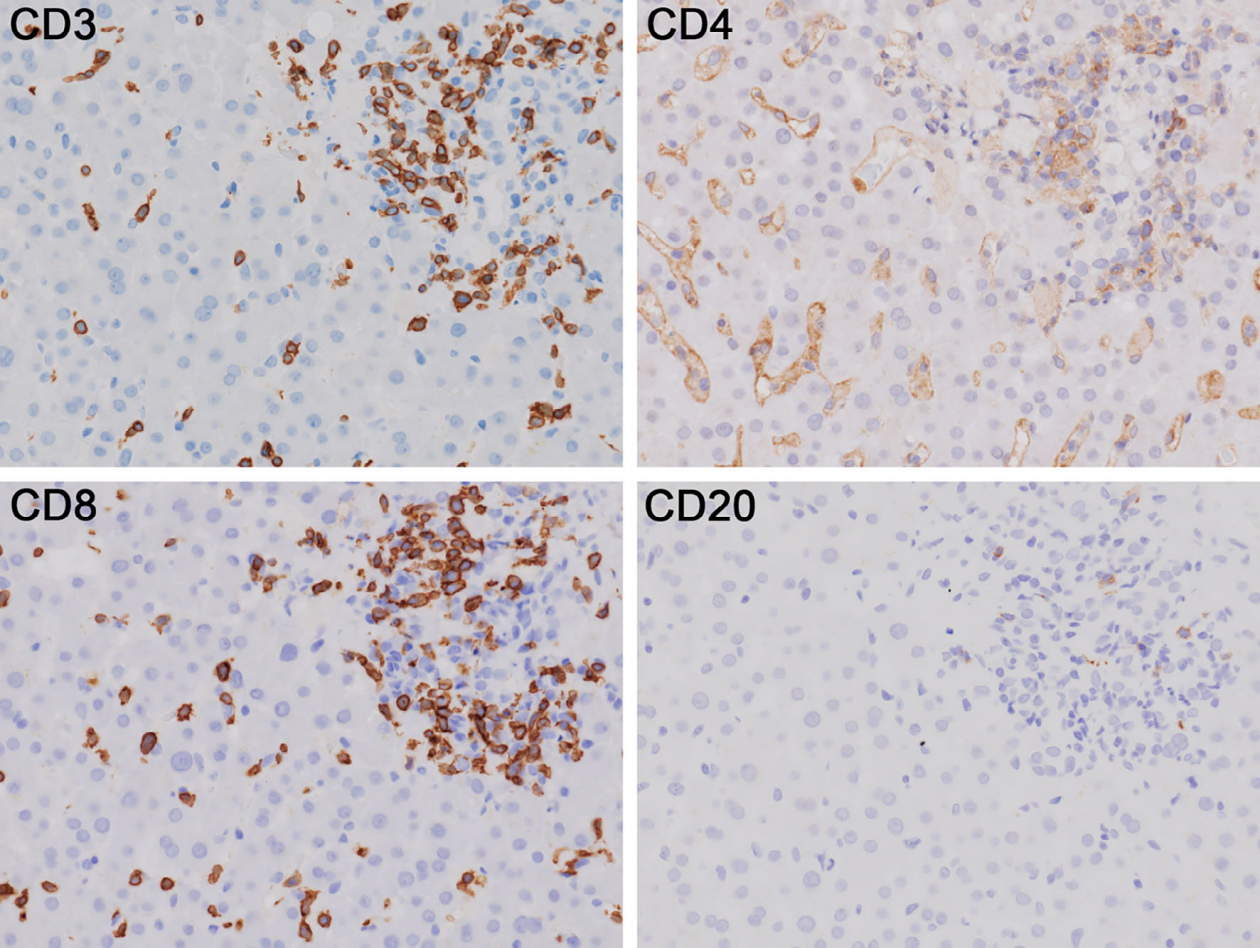
Immunohistochemical staining of liver specimens with liver injury resulting from osimertinib immediately after nivolumab therapy. In Case 4, CD3 and CD8 lymphocytes were predominantly expressed in the liver tissues compared to CD4 and CD20 lymphocytes.

Case 4, a 63‐year‐old male, was treated with osimertinib as a sixth line treatment on day 32 after nivolumab, exhibited the best response to PD. The antinuclear antibody (ANA) value before nivolumab was less than 40 titers. Although tumor shrinkage occurred following osimertinib treatment, he experienced serious hepatotoxicity (Fig 1). Grade 3 hepatotoxicity, accompanied by elevated alkaline phosphatase (grade 3), γ‐glutamyl transpeptidase, and total bilirubin (grade 3), was observed on day 29 after osimertinib. Subsequently, a diagnostic liver biopsy was performed to investigate liver dysfunction (Fig 2), for which the ANA value was less than 40 titers and antimitochondrial M2 antibody was negative. Additionally, serum immunoglobulin G and M levels were 1548 mg/dL and 112 mg/dL, respectively. Oral administration of prednisolone 120 mg (2 mg/kg) was started, followed by oral administration of mycophenolate mofetil 2 g/day, which gradually improved the hepatotoxicity. To investigate lymphocyte infiltration in severe liver dysfunction, liver biopsies were performed for different diagnoses in Cases 2 and 4. Relatively marked lymphocytic infiltration was histologically identified in the liver parenchyma and Glisson's capsules. However, bridging and confluent necrosis of the hepatocytes, bile duct injury, and plasma cell infiltrate were not observed. Immunohistochemically, CD3+ and CD8+ lymphocytes predominantly infiltrated the liver tissues, unlike CD20+ or CD4+ lymphocytes (Fig 2).

## Discussion

To the best of our knowledge, our retrospective study is the first to identify clinical aspects of serious hepatotoxicity related to osimertinib treatment immediately after nivolumab in patients with advanced NSCLC harboring the *EGFR* mutation.

Schoenfeld *et al*. reported that severe irAE occurred in six out of 41 patients (15%) who received osimertinib after ICIs, and one of the six patients developed grade 4 hepatotoxicity.^7^ In addition, it is known that hepatotoxicity is increased by molecular targeted therapeutic agents other than osimertinib and ICIs. In a phase 1/2 study (KEYNOTE‐021) evaluating the safety and tolerability of pembrolizumab in combination with erlotinib or gefitinib as a first‐line therapy in patients with advanced NSCLC harboring sensitizing *EGFR* mutation, grade 3 or 4 hepatotoxicity was observed in 71.4% of patients who received a combination of gefitinib and pembrolizumab.^8^ Furthermore, the phase 1/2 study (CheckMate 370) to evaluate the safety and tolerability of the combination of nivolumab and crizotinib in NSCLC patients with anaplastic lymphoma kinase (ALK) translocation reported that five of 13 patients (38%) who received nivolumab plus crizotinib developed grade 3 or 4 hepatotoxicity with therapeutic discontinuation, and two patients died of hepatotoxicity.^9^ Furthermore, a retrospective study of crizotinib for NSCLC with *ALK*, c‐ros oncogene 1 (*ROS‐1*), and *MET* gene abnormalities also reported a significant increase in hepatotoxicity after using crizotinib after ICIs.^10^ However, grade 3 or higher hepatotoxicity was not reported in the TATTON study using osimertinib and durvalumab,^3^ inconsistent with the results of this study. In the TATTON trial, durvalumab was coadministered with osimertinib, but in our study, osimertinib was administered immediately after nivolumab. The difference in the incidence of severe hepatotoxicity may be related to the difference in the method of administration.

In pooled analysis of clinical trials, hepatotoxicity of grade 3 or higher of the first‐ or second‐generation EGFR‐TKI was 18%, 5.4%, and 1.7% for gefitinib, erlotinib, and afatinib, respectively.^11^ In a prospective study, the frequency of grade 3 or higher hepatotoxicity by osimertinib was reported as 1%.^1^ As shown in our study, the frequency of hepatotoxicity with grade 3 or higher due to osimertinib immediately after ICI was 57.1%, which was significantly higher than the results of previous clinical trials using EGFR‐TKI.

Osa *et al*. developed a method to monitor the state of anti‐PD‐1 antibody binding to T lymphocytes. By this method, nivolumab was found to bind to T lymphocytes for more than 20 weeks, even after stopping treatment, suggesting a sustained therapeutic effect.^12^ Therefore, the synergistic effect of osimertinib and nivolumab may contribute to the high incidence of hepatotoxicity.

In this study, the liver biopsy tissues of Cases 2 and 4 showed marked infiltration of CD8 + lymphocytes into hepatocytes. In the cases in our study with severe hepatotoxicity, T lymphocytes were proven to infiltrate the liver tissues compared to B lymphocytes. After confirming severe hepatotoxicity, whether there are any infiltrations of neutrophils, eosinophils, and lymphocytes within the liver tissues should be determined. Considering their association with ICIs, the role of lymphocytes infiltrating liver tissues in liver toxicity should be emphasized for the differential diagnoses. Zen *et al*. compared the pathological characteristics of autoimmune hepatitis (AIH) and hepatitis due to ICI^13^ and evaluated the histological findings of liver injury after ICI treatment in seven patients (nivolumab in five and ipilimumab in two patients). They demonstrated that centrilobular confluent necrosis and plasmacytosis in hepatitis relevant to ICI were less common and milder than those in AIH. Furthermore, they immunohistochemically revealed the presence of numerous CD3+ and CD8+ lymphocytes, whereas ICI‐associated hepatitis contained fewer CD20+ plasma cells and CD4+ T cells than AIH. Based on these histopathological findings, there remains a high possibility of drug‐induced liver injury in our current case, accompanied by the serological findings and clinical course. Moreover, predominant infiltration of CD3 and CD8 in the liver specimens, rather than CD4 and CD20, strongly suggests immunogenic activation of T cell lymphocytes due to nivolumab. Considering the results of our retrospective analysis, serious hepatotoxicity resulting from osimertinib immediately after ICI treatment may occur in relation to the activation of lymphocytes by the administration of nivolumab, supported by our immunohistochemical findings. As there was a significant difference in the incidence of grade 3 or 4 hepatotoxicity between the DSG and non‐DSG groups, the frequency of this toxicity may differ according to the period from the last administration of nivolumab to the initiation of osimertinib. Generally, the administration of osimertinib as a third generation EGFR‐TKI immediately after ICI treatment is not permitted because of a high incidence of ILD; however, as the first‐ or second‐generation EGFR‐TKI immediately after ICI is described to be feasible without increased frequency of ILD.^5^ First generation, especially erlotinib or second‐generation EGFR‐TKI, can be administered without severe hepatotoxicity, even immediately after ICI treatment.

There were some limitations to this study. First, our approach was a retrospective assessment; moreover, our sample size was small. Therefore, this may bias the results of our study. As the sequence treatment of osimertinib immediately after ICI should be prohibited, this should be confirmed by a further prospective study or a study with a large sample size. Second, diagnostic liver biopsy was not performed in all patients who experienced severe hepatotoxicity. In two cases who underwent liver biopsy, CD3 and CD8 lymphocytes were predominantly expressed in the liver tissues with hepatotoxicity. Considering the results of liver specimens, a synergistic effect of osimertinib and change in immune environment may contribute to the increased hepatotoxicity. Further investigations using additional cases might clarify the significance of their clinical and pathological conditions. Finally, little is known about the detailed mechanism how immunotherapy could cause serious hepatotoxicity, triggered by EGFR‐TKI. Thirdigeneration EGFR‐TKI may work as a strong trigger causing severe toxicities after immunotherapy. Although we found that a synergistic effect of osimertinib and nivolumab is closely linked to the increased incidence of severe hepatotoxicity, its mechanism was not proven in our study. Further studies are warranted to elucidate the mechanism of adverse events related to EGFR‐TKI after administration of immunotherapy.

In conclusion, the use of osimertinib immediately after nivolumab is likely to increase the frequency of grade 3 or 4 hepatotoxicity. Therefore, physicians should design treatment sequences that consider the risk of hepatotoxicity.

## Disclosure

OY received speaker honoraria from Ono Pharmaceutical Co., Ltd., Bristol‐Myers Squibb, Taiho Pharmaceutical, MSD, Chugai Pharmaceutical Co., Ltd., and AstraZeneca. KK received speaker honoraria from Ono Pharmaceutical Co., Ltd., Bristol‐Myers Squibb, Taiho Pharmaceutical, Chugai Pharmaceutical Co., Ltd., Eli Lilly Japan, and AstraZeneca. AM received speaker honoraria from Ono Pharmaceutical Co., Ltd., Bristol‐Myers Squibb, Taiho Pharmaceutical, MSD, Chugai Pharmaceutical Co., Ltd., and AstraZeneca. KK received research grants from AstraZeneca and Bristol‐Myers Squibb. KK received speaker honoraria from AstraZeneca, Pfizer Japan Inc., Ono Pharmaceutical Co., Ltd., Chugai Pharmaceutical Co., Ltd., and Bristol‐Myers Squibb. SM received lecture fees from Abbvie GK, Gilead Sciences Inc., MSD K.K., Ohtsuka Pharmaceutical Co. Ltd., Bristol‐Myers Squibb Co., Dainihon‐Sumitomo Pharma Co. Ltd., Asuka Pharmaceutical Co. Ltd., Torey Medical, Asahikasei Pharma Co., and Kyowa Hakko Kirin Co. Ltd. SM received consigned/joint research expenses from Gilead Sciences Inc., EA Pharma Co., Ltd., Janssen Pharmaceutical K.K., Kowa Co. Ltd., MSD K.K., and Abbvie GK. SM received Scholarship donations from Abbvie GK, MSD K.K., Dainihon‐Sumitomo Pharma Co. Ltd., Mochida Pharmaceutical Co. Ltd., Daiich Sankyo Co. Ltd., Torey Medical, Chugai Pharmaceutical Co., SRL Inc., EA Pharma Co., Ltd., and Japan Blood Products Organization. HK received research grants from Novartis Pharma K.K., Nippon Boehringer Ingelheim Co., Ltd., Chugai Pharmaceutical Co., Ltd., Taiho Pharmaceutical, and Ono Pharmaceutical Co., Ltd. HK received speaker honoraria from Chugai Pharmaceutical Co., Ltd., AstraZeneca, Pfizer Japan Inc., Eli Lilly Japan, Novartis Pharma K.K., Nippon Boehringer Ingelheim Co., Ltd., Ono Pharmaceutical Co., Ltd., Bristol‐Myers Squibb Co., Taiho Pharmaceutical, and MSD. TK, KH, AS, SS, YM, FN, and YM declare no conflicts of interest.
